# Determinants of healthcare seeking and out-of-pocket expenditures in a “free” healthcare system: evidence from rural Malawi

**DOI:** 10.1186/s13561-020-00271-2

**Published:** 2020-05-27

**Authors:** Meike Irene Nakovics, Stephan Brenner, Grace Bongololo, Jobiba Chinkhumba, Olivier Kalmus, Gerald Leppert, Manuela De Allegri

**Affiliations:** 1 Heidelberg Institute of Global Health, Faculty of Medicine and University Hospital, University of Heidelberg, Heidelberg, Germany; 2 Research for Equity and Community Health (REACH) Trust, Lilongwe, Malawi; 3 University of Malawi College of Medicine, Blantyre, Southern Region Malawi; 4 German Institute for Development Evaluation (DEval), Bonn, Germany

**Keywords:** Health care seeking behaviour, Health financing, Costs, Health care allocation, (country of expertise: Malawi)

## Abstract

**Background:**

Monitoring financial protection is a key component in achieving Universal Health Coverage, even for health systems that grant their citizens access to care free-of-charge. Our study investigated out-of-pocket expenditure (OOPE) on curative healthcare services and their determinants in rural Malawi, a country that has consistently aimed at providing free healthcare services.

**Methods:**

Our study used data from two consecutive rounds of a household survey conducted in 2012 and 2013 among 1639 households in three districts in rural Malawi. Given our explicit focus on OOPE for curative healthcare services, we relied on a Heckman selection model to account for the fact that relevant OOPE could only be observed for those who had sought care in the first place.

**Results:**

Our sample included a total of 2740 illness episodes. Among the 1884 (68.75%) that had made use of curative healthcare services, 494 (26.22%) had incurred a positive healthcare expenditure, whose mean amounted to 678.45 MWK (equivalent to 2.72 USD). Our analysis revealed a significant positive association between the magnitude of OOPE and age 15–39 years (*p* = 0.022), household head (*p* = 0.037), suffering from a chronic illness (*p* = 0.019), illness duration (*p* = 0.014), hospitalization (*p* = 0.002), number of accompanying persons (*p* = 0.019), wealth quartiles (*p*_2_ = 0.018; *p*_3_ = 0.001; *p*_4_ = 0.002), and urban residency (*p* = 0.001).

**Conclusion:**

Our findings indicate that a formal policy commitment to providing free healthcare services is not sufficient to guarantee widespread financial protection and that additional measures are needed to protect particularly vulnerable population groups.

## Background

In April 2018, the World Health Organization (WHO) celebrated the 70th anniversary of World Health Day and the 40th anniversary of the Alma Ata Declaration, placing the year 2018 under the theme “*Universal Health Coverage (UHC): Everyone, Everywhere*” [1]. The concept of UHC sits at the core of Sustainable Development Goal 3 [2]. It reminds the international community of the basic human right to health, and calls upon nations to implement health systems that secure access to quality care while ensuring financial protection against the cost of illness.

In contrast to the current movement towards UHC, in the 1980s and 1990s many African countries were under pressure to implement Structural Adjustment Policies [3] and introduced user charges for healthcare services [4, 5], which was supported by the Bamako Initiative [6]. Malawi is an example of a country that resisted the push to finance healthcare provision through the application of user fees. Shortly after independence it implemented a free healthcare system (financed primarily by a combination of government and donor funds) to ensure that access to health services in public facilities would not be conditional upon user charges [7–9]. In 2004, the government of Malawi further refined free healthcare provision with the introduction of an explicit Essential Health Package (EHP), clearly stipulating which services were to be provided free of charge at point of use in public and in contracted private health facilities [10, 11].

Prior evidence suggests that despite the formal stipulations of government policies, important barriers to adequate health service utilization for people in need persist. Specifically, the existing literature has identified the presence of financial barriers imposed by informal payments and travel costs [12–15]; quality of care barriers, related to drug stock-outs, chronic shortage of highly-qualified staff, and users’ lack of information on probable medical benefits [9, 13–18]; distance to health facilities, due to an uneven geo-spatial distribution of healthcare providers across regions [13, 15, 17]; and cultural barriers, related to low educational levels, traditional beliefs, and fear of stigma [12, 13, 15, 16].

In particular, prior evidence suggests that due to funding shortages and inefficiencies in health service delivery, Malawians are still exposed to considerable out-of-pocket expenditures (OOPE) when seeking care, even in public facilities [19–22]. OOPE deter health service utilization, especially among the very poor, given the prospect of having to pay substantial amounts to receive care [14, 16, 20, 22, 23], while also imposing a considerable financial burden on those who seek treatment [22, 24]. Moreover, additional evidence from Malawi indicates that current levels of OOPE often lead to catastrophic expenditure [25, 26] resulting in considerable impoverishment rates [22, 27].

This substantial literature on OOPE suffers from two weaknesses, however: an exclusive focus on certain populations [20, 22] and/or certain conditions [20–24], and the application of statistical methods that do not account for the peculiarity of modelling health expenditure data. More specifically, modelling OOPE needs to account for the selection bias that emerges as a consequence of the fact that a positive expenditure can only be observed for those who decide to seek care in the first place [28–31].

In line with current prescriptions to monitor financial protection as an integral component of assessing progress towards UHC [32, 33], our study set as its primary objective the estimation of OOPE for curative services and their determinants in rural Malawi. In pursuing our objective, we made an explicit effort to go beyond existing literature. Hence, we relied on population-based data to assess OOPE for curative services across a wide range of conditions and population groups and applied a methodology, the Heckman selection model, that accounts for the bias that arises from observing OOPE only for those individuals who sought formal healthcare services in the first place. Given that accounting for selection bias inevitably relies on first estimating service use, our study addresses as secondary objective determinants of utilization of formal healthcare services among the same rural population.

## Method

### Study setting

With a gross national per capita income of 1064 PPP USD [34], the landlocked sub-Saharan African (SSA) country Malawi is ranked 171th out of 189 countries on the 2017 Human Development Index [34, 36]. 71.4% of the population live under the poverty line of 1.90 PPP USD per day [35]. Nearly 80% of the population live in rural areas and rely primarily on subsistence farming [36]. The country is affected by a high degree of morbidity and mortality, mainly due to malnutrition and infectious diseases such as HIV/AIDS, malaria, acute respiratory infections, stroke and diarrhea [37, 38]. In addition, chronic conditions such as asthma, cancer, high blood pressure, cardiovascular diseases, and diabetes are on the rise, imposing an additional challenge to effective service provision in an already strained healthcare system [36, 39–41].

Healthcare provision is organized in a three-tier system, with health centers, community hospitals, dispensaries, and maternity units at the primary level serving as the first point of contact for most patients; district hospitals equipped with basic surgical facilities providing secondary care; and central hospitals located in the four largest cities of the country providing tertiary care. As described earlier, government-owned health facilities and selected private facilities (for example, those of the Christian Health Association (CHAM)) contracted by the Ministry of Health via Service Level Agreements (SLAs) are expected to provide EHP services with no fees at point of use. In principle, the EHP includes a wide range of cost-effective services for the prevention and treatment of communicable and non-communicable diseases, malnutrition, and maternal and perinatal conditions [42]. Approximately 60% of all health facilities in Malawi belong to the government, 36% to the CHAM, and the remaining 4% belong to private for-profit providers [11, 37, 43].

In 2014, total per capita health expenditure amounted to 93 PPP USD per year, equivalent to 11.4% of GDP [38]. Of this amount, donor funding accounted for 74%, domestic funding accounted for 19%, and OOPE for 7% [44].

### Data sources

This study used data from the first (August to October 2012) and the second (March to May 2013) round of a household survey conducted in three districts in rural Malawi (Chiradzulu, Thyolo, Mulanje) and was initially set up to evaluate the impact of a micro-health insurance scheme planned, but never implemented, by the largest Malawian micro-finance organization, MUSCCO. Details of the survey have been described before [45]. In brief, data were collected on a total sample of 1639 households selected across 114 villages using a two-stage sampling procedure. Approval was granted by the relevant ethical committees.

The questionnaire collected information on households’ demographic and socio-economic profiles as well as on individual’s acute and chronic illness reporting, health care seeking behavior, including use of both formal (i.e., Western facility-based care) and informal (i.e., traditional healers, community health workers, pharmacies) health services, and related OOPE (including transport). We categorized pharmacies, community health workers and community nurses as non-formal care because, at the time of data collection, these two categories were not part of any formal curative healthcare provision program. Information from individual household members were collected by trained research assistants using a digitalized data entry system. Mothers or primary caretakers acted as proxy respondents for children below the age of 14, while households determined whether individuals aged 14 to 17 years should respond on their own or not.

### Variables and their measurement

Table 1 contains a list of all variables included in this study, their measurement, and the expected sign of the association with OOPE. Statistics about health expenditures are listed in Table 3. Most of the variables listed in Table 1 are self-explanatory.

**Table 1:** Variables, their measurements, and hypothesized sign of the association with out-of-pocket expenditure on medical care at formal healthcare facilities

	Measurement and categorization	Expected direction of relation to OOPE
**Outcome variables**		
Out-of-pocket expenditures (OOPE) on medical treatment	Continuous	
Utilization of formal healthcare services (in the last 4 weeks)	0 = no care or informal care (incl. Self-care; community health worker or community nurse; traditional healer or herbalist) 1 = formal care (incl. Visit to either a public, a private or a not-for-profit Western healthcare facility)	
**Selection variable**		
Distance to the closest health facility (km)	Continuous (measured as straight-line distance)	
**Explanatory variables**		
Age	0 = 0–4 years 1 = 5–14 years 2 = 15–39 years 3 = 39+ years	+
Sex	0 = male 1 = female	–
Education (education status of household head if children < 14 years)	0 = no formal education 1 = any formal education	+
Household head	0 = other 1 = being household head	+
Reported chronic illness	0 = no chronic illness reported 1 = chronic illness reported	+
Illness duration (days)	Continuous	+
Limitation imposed on routine activities	0 = no perceived limitation on routine activities 1 = perceived limitation on routine activities	+
Hospitalization	0 = no hospitalization 1 = hospitalization	+
Accompanying persons	Continuous	+
Socio economic status (wealth quartiles)	1 = poorest (1st wealth quartile) 2 = poor (2nd wealth quartile) 3 = less poor (3rd wealth quartile) 4 = least poor (4th wealth quartile)	+
Household size	0 = 0–5 members 1 = 5+ members	–
Location of household	0 = rural 1 = urban	+

#### Outcome variables

In line with our objective to explore the extent to which direct payments at point of use persist within the framework of a free healthcare system, we defined OOPE (i.e., individual nonzero healthcare expenditures) for people seeking care at formal healthcare facilities as our primary outcome. In line with the abovementioned definition of OOPE, we defined having sought care at a formal healthcare facility as our outcome for the selection model. More specifically, we focused on the sub-sample of individuals reporting at least one acute illness episode over the course of the prior 4 weeks and distinguished individuals who sought formal care at a health facility (coded as 1) from individuals who visited a community health worker or community nurse, a traditional healer or herbalist, or who did not seek care at all (coded as 0).

Our OOPE variable included only consultation and treatment expenses, such as expenses for laboratory tests (X-rays etc.), drugs (tablets, injections, infusions, topical preparations etc.), medical devices (crutches, glasses etc.), and any additional formal or informal fees paid. In spite of being aware that transportation expenses represent an important component of the financial burden imposed on households in rural African settings [46–48], we excluded them from the computation of our OOPE variable, since our objective was to estimate the financial protection granted specifically by the Malawian free healthcare policy, and at the time of study (and until today), there is no direct provision to include travel expenses in the EHP. Still, we report transportation expenses separately to provide a comprehensive picture of the financial burden illness episodes impose on Malawian households. A detailed listing of the different medical expenses for medication, laboratory etc. was not available.

#### Selection variable

We adopted *distance to the nearest healthcare facility*, measured as a straight-line distance using GPS coordinates [23, 49], as the selection variable since prior studies in Malawi [21, 50] and elsewhere in Sub-Saharan Africa [51, 52] have identified it as a major determinant of healthcare seeking, but not of OOPEs on medical treatment. Accordingly, we did not include distance as an explanatory variable in our primary model, but only in our selection model.

#### Explanatory variables

Explanatory variables were selected on the basis of prior evidence indicating their association with OOPE [21, 23, 53] and of pragmatic considerations regarding availability in the specific database at our disposal.

We divided *age* into four groups in alignment with the defined ages rages for child labor [54] to facilitate the interpretation of our findings for policy purposes. We included a measure of *education*, assuming that better educated individuals are generally more empowered to make decisions on their own or their dependents’ health and may therefore face higher OOPE on medical treatment. We assigned the educational status of the household head to children under 14, since we assumed that they would be the ones mediating the decision to seek care for minors [16, 55]. We determined whether the individual reporting an illness was the *household head* himself/herself, because prior research indicates a higher propensity to seek formal care [56] and incur higher OOPE [20, 23, 57].

We included a measure of whether the individual reported any *chronic illness*, defined as any condition a person suffered from for more than 3 months and not restricting it only to non-communicable diseases as done in previous studies [23]. We postulated that individuals with an underlying chronic condition might be exposed to important co-morbidities and hence face higher OOPE.

Similarly, we assumed that individuals might face higher OOPE for more severe conditions, and therefore included three different measures of illness severity: self-reported *illness duration*, resulting *degree of limitation imposed on routine activities,* and *hospitalization.* Further, we determined the number of *accompanying persons* who assisted an ill individual when seeking care, since, based on prior qualitative evidence also from Malawi [55], we postulated that individuals affected by more severe conditions may require additional support in seeking care and, as a consequence, may incur higher OOPE.

*Household wealth* quartiles were used as proxy of household socio-economic status. We computed a wealth index by aggregating information on physical household infrastructure, durable assets, and owned animals using Multiple Correspondence Analysis [58, 59]. In line with prior literature [21, 22], we hypothesized that, given a higher capacity to pay, better off individuals would face higher OOPE than poorer individuals. We also included a measure of *household size*, since we assumed that decisions on intra-household resource allocation might be more complex for larger households resulting in lower ability to pay for the single individual member.

We included the *location of the household*, since we assumed that in urban settings individuals might incur higher OOPE due to a greater availability of diagnosis and treatment options, in line with previous findings [21, 22, 60]. It ought to be noted, however, that in our study urban setting only refers to small district towns and not to cities such as Lilongwe or Blantyre.

It also ought to be noted that we would have liked to differentiate OOPE for people seeking care at public vs. private (including not-for-profit) facilities, but unfortunately information on facility ownership was missing for over two-thirds of our sample, possibly suggesting that individuals may not be able to recall facility ownership.

### Analytical approach

Prior to beginning our analysis, we pooled all illness episodes detected in the 2012 and in the 2013 survey rounds into a single sample. Then, using the pooled sample, we relied on descriptive statistics to identify a sample distribution for all variables included in our study. We calculated mean, standard deviation (SD), median, and range values for OOPE on medical treatment (our primary outcome) and for expenditure on transport. To account for the heavily right-skewed distribution of OOPE [61], we used boxplots to detect outliers [62, 63]. To handle the outliers, we used winsorization, since truncation or trimming can lead to substantial bias for resulting mean values [61]. Accordingly, we replaced the upper 5% of outliers with the respective highest value of the sub-sample without outliers (i.e., 95% percentile value) [64]. This approach allowed us to keep the entire sample while avoiding a situation where extreme outliers would distort the findings of the regression analysis [61, 62, 64].

Last, we relied on a Heckman selection model to identify the determinants of OOPEs on medical treatment conditional upon having sought formal care at a healthcare facility. We relied on a Heckman selection model rather than standard linear regression, since the outcome of interest, OOPE on medical treatment, could only be observed for individuals who sought care at a formal health facility in the first place [28, 30]. Through the application of a two-step statistical approach, the Heckman model offers a means of correcting for non-random samples. In the Heckman model, OOPE on medical treatment for individual i with the attributes *x*_*i*_ is defined as (primary equation):

$$ {OOPE}_i=\kern0.5em {x}_i\kern0.5em \beta \kern0.5em +\kern0.5em {\in}_i $$

under the condition that the individual sought care at a formal health facility (selection equation):

$$ {z}_i\kern0.5em \gamma \kern0.5em +\kern0.5em {x}_i\kern0.5em \partial \kern0.5em +\kern0.5em {\nu}_{i\kern0.5em }\kern0.5em >0 $$

where *β* and *∂* are the coefficients of the attributes in the primary and in the selection equation. The selection variable is *z*_*i*_ (here the distance to the nearest health facility), *γ* its coefficient and the following applies:

$$ {\in}_i\kern0.5em \sim \kern0.5em N\left(0,\sigma \right)\kern0.5em and\kern0.5em {v}_{i\kern0.5em }\kern0.5em \sim \kern0.5em N\left(0,1\right)\kern0.5em and\kern0.5em corr\left({\in}_i,{v}_i\right)\kern0.5em =\kern0.5em \rho $$

This means that if there is no self-selection effect (*ρ* = 0), the selection and the regression equation can be analyzed separately. In our case, however, we could not reject the null-hypothesis and could identify a selection effect, which could be effectively accounted for by the selection variable, i.e., distance to the nearest healthcare facility (Wald test of independence significant on level 1% and selection variable significant on level 5%). To take possible intra-correlation on individual level into account, robust standard errors (SE) were estimated. Data analysis was performed using STATA 14.

## Results

Across the two survey rounds, we identified a total of 2740 acute illness episodes (over the prior 4 weeks) distributed across 1988 individuals in 1037 households. Table 2 reports the basic socio-demographic and economic characteristics of individuals reporting acute illness episodes, of the sub-sample of episodes making use of formal healthcare services, and of the sub-sample of episodes incurring a positive OOPE on medical treatment upon making use of such services. The average age of individuals reporting an illness episode and seeking care at a formal facility was 19.56 years (SD 17.57 years). Of those reporting an illness episode and seeking care at a formal facility, 55.20% were women, 61.78% had no formal education, and 10.93% reported a chronic co-morbidity.

**Table 2:** Sample characteristics

		Acute ill sample (***n*** = 2740)		Utilization formal care sample (***n*** = 1884)		Positive out-of-pocket expenditures on medical treatment sample (***n*** = 494)	
**Sample scale**		**N**		**N**		**N**	
	Number of villages	101		98		75	
	Number of households	1037		718		186	
**Individual level**							
		**Mean**	**SD**	**Mean**	**SD**	**Mean**	**SD**
Distance to the closest health facility (km)	continuous	2.22	1.25	2.19	1.24	2.14	1.28
Age (years)	continuous	21.43	18.2	19.56	17.57	19.62	17.12
Illness duration (days]	continuous	7.91	8.27	8.67	8.85	9.58	10.21
Accompanying persons	continuous			1.19	0.61	1.22	0.60
		**N**	**%**	**N**	**%**	**N**	**%**
Age	0 = 0–4 years;	513	18.72	419	22.24	117	23.68
	1 = 5–14 years;	793	28.94	562	29.83	133	26.92
	2 = 15–39 years;	971	35.44	637	33.81	171	34.62
	3 = 39+ years	463	16.90	266	14.12	73	14.78
Sex	0 = male;	1283	46.82	844	44.80	230	46.56
	1 = female	1457	53.18	1040	55.20	264	53.44
Education (education of household head if child < 14 years)	0 = no formal education 1 = any formal education	1726 1014	62.9937.01	1164 720	61.78 38.22	295 199	59.72 40.28
Household head	0 = others;	2157	78.72	1523	80.84	391	79.15
	1 = being household head	583	21.28	361	19.16	103	20.85
Reported chronic illness	0 = no chronic illness reported;	2356	85.99	1678	89.07	436	88.26
	1 = chronic illness reported	384	14.01	206	10.93	58	11.74
Limitation imposed on routine activities	0 = no perceived limitation on routine activities	1047	38.21	587	31.16	125	25.30
	1 = perceived limitation on routine activities	1693	61.79	1297	68.84	369	74.70
Hospitalization	0 = no hospitalization;	2636	96.20	1780	94.48	463	93.72
	1 = hospitalization	104	3.80	104	5.52	31	6.28
Socio economic status (wealth quartiles)	1 = poorest (1st wealth quartile)	686	25.04	484	25.69	108	21.86
	2 = poor (2nd wealth quartile)	685	25.00	486	25.80	109	22.06
	3 = less poor (3rd wealth quartile)	685	25.00	478	25.37	126	25.51
	4 = least poor (4th wealth quartile)	684	24.96	436	25.14	151	30.57
Household size	0 = 0–5 members;	1483	54,12	1036	54.99	297	60.12
	1 = 5+ members	1257	45,88	848	45.02	197	39.88
Location of household	0 = rural	2598	94.82	1791	95.06	470	95.14
	1 = urban	142	5.18	93	4.94	24	4.86

*SD* Standard Deviation

Approximately one fourth of all illness episodes treated at a formal facility (n = 494, 26.22%) generated a positive OOPE on medical treatment with a mean of 678.45 MWK (SD = 758.63 MWK; Table 3), equivalent to 2.72 USD (all amounts in USD are calculated on basis of the conversion rate of 249.11 MWK = 1 USD at the time of data collection [65]). In addition, 403 (21.39%) illness episodes treated at a formal facility generated transportation costs, with a mean of 516.13 MWK (SD = 458.55 MWK), equivalent to 2.07 USD.

**Table 3:** Individual out-of-pocket expenditure^b^ on medical treatment among individuals seeking care at a healthcare facility (*n* = 1884; zeros excluded)

	N	%	Mean^**a**^	SD^**a**^	Median^**a**^	Min^**a**^	Max^**a**^
**Out-of-pocket expenditures on medical treatment**^**c**^	494	26.22	678.45	758.63	350	10	2500
**Transportation expenditure**	403	21.39	516.13	458.55	400	20	2000

*SD* Standard Deviation, *MWK* Malawian Kwacha

^a^Values are expressed in MWK (249.11 MWK = 1 USD at time of data collection (World Bank, 2019))

^b^Winsorized direct costs (excluding zeros): Replacement of the right outliners with the 95th percentile (Facility healthcare expenditures) or the 95th percentile (transport expenses)

^c^Out-of-pocket expenditures on medical treatment for formal care include all consultation and treatment expenses, including: laboratory tests (x-rays etc.), drugs (tablets, injections, infusions, topical preparations etc.), medical devices (crutches, glasses etc.), as well as informal fees

The analysis of the selection equation (Table 4) indicates that increasing distance to the health facility (coeff = − 0.090; *p* = 0.015), increasing age (coeff_2_ = − 0.509; *p*_2_ < 0.001; coeff_3_ = − 0.722; *p*_3_ < 0.001; coeff_4_ = − 0.973; *p*_4_ < 0.001), having a chronic illness (coeff = − 0.353; *p* = 0.004), and a household size over five members (coeff = − 0.212; *p* = 0.022) significantly decreased the likelihood of utilizing formal care. In contrast, being female (coeff = − 0.172; *p* = 0.076), illness duration (coeff = 0.031; *p* < 0.001), incurring limitations in routine activities (coeff = 0.746; *p* < 0.001), being hospitalized (coeff = 7.006; *p* < 0.001), and belonging to the highest wealth quartile (coeff = 0.234; *p* = 0.081) significantly increased the likelihood of utilizing formal healthcare.

**Table 4:** Heckman model for determinants of utilization formal care (*n* = 1884) and of OOPE on medical treatment (*n* = 494) run on a sample of 2740 acute ill episodes

	First part selection eqn.: Utilization of formal healthcare services (in the last 4 weeks)			Second part primary equation: Out-of-pocket expenditures^**1**^ on medical treatment		
Explanatory variables	Coeff	SE^2^	*P*-value	Coeff	SE^2^	*P*-value
Distance to the closest health facility (km)	−0.090**	0.037	0.015			
Age						
5–14 years	−0.509***	0.140	< 0.001	98.934	77.943	0.25
15–39 years	−0.722***	0.145	< 0.001	238.548**	99.862	0.017
39+ years	−0.973***	0.187	< 0.001	210.440	166.323	0.21
Female	0.172*	0.097	0.076	−56.623	72.883	0.44
Any formal education	0.109	0.100	0.28	−18.193	78.430	0.82
Being household head	0.170	0.141	0.23	304.119**	146.076	0.037
Chronic illness reported	−0.353***	0.121	0.004	286.869**	122.242	0.019
Illness duration (days)	0.031***	0.006	< 0.001	9.838**	4.013	0.014
Limitation imposed on routine activities	0.746***	0.094	< 0.001	−56.257	77.537	0.47
Hospitalization	7.006***	0.226	< 0.001	713.743***	235.554	0.002
Accompanying persons	0.016	0.083	0.85	162.949**	69.195	0.019
Socio economic status (wealth quartiles)						
Poor (2nd wealth quartile)	−0.166	0.133	0.21	213.333***	90.557	0.018
Less poor (3rd wealth quartile)	0.080	0.133	0.55	322.699***	101.033	0.001
Least poor (4th wealth quartile)	0.234*	0.134	0.081	321.486***	102.957	0.002
Household size 5+ members	−0.212**	0.093	0.022	−75.62657	72.028	0.30
Urban	−0.126	0.196	0.52	692.753***	208.922	0.001

Wald test of indep. Eqns. (rho = 0): chi2 (1) = 11.96***

Prob > chi2 = 0.0005

*eqn.* equation, *Coeff* Coefficient, *SE* standard error

*** Significant at 1%, ** significant at 5%, * significant at 10%

^1^Out-of-pocket spending includes facility healthcare costs

^2^Robust SE adjusted for individual level

The results of the primary equation (Table 4) show that OOPE on medical treatment were significantly higher for individuals aged 15–39 years (coeff = 238.548; *p* = 0.017), household heads (coeff = 304.119; *p* = 0.037), individuals reporting chronic illness (coeff = 286.869; *p* = 0.019), individuals with longer illness duration (coeff = 9.838; *p* = 0.014), individuals who had been hospitalized (coeff = 713.743; *p* = 0.002), individuals requiring more accompanying persons (coeff = 162.949; *p* = 0.019), individuals from higher socio economic strata (coeff_2_ = 213.333; *p*_2_ = 0.018; coeff_3_ = 322.699; *p*_3_ = 0.001; coeff_4_ = 321.486; *p*_4_ = 0.002), and individuals living in urban areas (coeff = 692.753; *p* = 0.001).

## Discussion

The study makes an important contribution to the existing literature monitoring progress towards UHC by assessing the extent to which the free healthcare system in place in Malawi effectively grants rural populations financial protection. Our findings indicate that in spite of the free healthcare policy, about one fourth of all individuals seeking care at a healthcare facility (conditional upon being ill) incurred a positive OOPE on medical treatment, ranging from 350 to 2500 MKW (Malawian Kwacha), with an average approaching 700 MKW (249.11 MWK = 1 USD [65]). In addition to paying for medical care, about one fifth of all respondents also incurred substantial travel expenses, ranging from 400 to 2000 MKW, with an average approximating 500 MKW. Our findings further indicate that productive age (15–39 years old), suffering from a chronic condition, being a household head, illness severity (proxied by hospitalization and need for a caregiver), wealth, and urban residency were all factors positively associated with the magnitude of OOPE on medical care.

While the proportion of people who incurred OOPE on medical treatment as well as the absolute value may initially appear low, one needs to appraise both values in relation to the prior evidence on financial protection in the health sector and the overall socio-economic reality of the country. First, one needs to consider that earlier studies have consistently reported the persistence of OOPE in spite of a healthcare system that in principle should afford free healthcare access [48, 66–68] and have attributed this persistence to health system failures related to shortages in drugs and lack of staff [66, 67]. The fact that our study also identifies OOPE suggests that there has been no progress in increasing financial protection in the health sector. Second, considering that 71% of the Malawian population lives on less than 1.90 USD per day [35], an expenditure on medical treatment for a single illness episode of 2.72 USD can easily impose a substantial financial burden, especially upon the poorest. Our findings indicate that the government urgently needs to implement concrete strategies to ensure that the free healthcare policy stipulated on paper and recently re-affirmed in the Health Sector Strategic Plan II [69] is effectively translated into practice. This means devoting sufficient resources to ensure continuity in staff presence and drug availability for all services included in the EHP. Additionally, the government is called to expand Service Level Agreements with private providers to ensure geographical accessibility to healthcare services for all of its citizens [9, 12, 70].

The fact that three-fourths of the people seeking formal healthcare services did not incur any expenditure could initially be taken as an indication of the fact that the free healthcare system is working in a relatively effective manner. Looking deeper into the data, and more specifically at the results of the selection model, however, indicates that only about two out of three illness episodes were handled at a healthcare facility, with greater distance being associated with a reduced probability of seeking care. In line with exiting literature of SSA, other factors associated with a reduced probability of seeking care were increasing age [71–73], suffering from a chronic condition [74], and a larger household size [75]; while all proxies for illness severity [45, 46, 76, 77], wealth [45, 72, 74, 78], and being female [47, 79, 80] were associated with an increased probability of seeking care. While the focus of this paper is on financial protection, acknowledging these barriers to access is nevertheless relevant from a policy perspective, since, in the absence of relevant data, we cannot exclude the possibility that the people who forewent seeking care at a healthcare facility did so out of fear of facing an expenditure. In addition, we do not have means to know whether the individuals who incurred no OOPE effectively received the full treatment they needed, including drugs, with no payment or simply forwent purchasing certain items due to lack of financial means.

One element noteworthy of the reader’s attention when jointly appraising results from our selection model and from our primary model is the fact that upon reporting ill, children under five and women appeared to be the most likely to seek care at a health facility, but not more likely to incur a larger expenditure. These findings stand in contradiction with evidence emerging from other settings suggesting that older individuals are the ones to be more likely to seek care [72, 78, 81–83] and with evidence suggesting that women are subject to consult a male in the household before making decisions on seeking care for themselves and their children [16, 55]. Our findings may suggest that the focus placed on achieving Millennium Development Goals 4 and 5 (targeting respectively child and maternal health) [84] effectively worked to encourage and enable early healthcare seeking among children under five and women by ensuring free or low cost healthcare provision for services targeting these vulnerable groups [85]. Further studies are needed to confirm this emerging hypothesis.

Our specific findings on the determinants of OOPE are not surprising, since they are well-aligned with prior evidence from other settings in SSA. In line with prior studies, our findings indicate higher OOPE for productive age groups [21, 82, 83]; for household heads [23]; for more severe illness episodes, proxied by longer illness duration and by the inability to carry out daily activities, by hospitalisation, and by number of accompanying persons [22, 23, 46, 86], for urban residency [21, 22, 83]; and increasing wealth [20–23, 83, 87–89].

Of particular interest in our study are the large coefficients suggesting that individuals who are essential for the household wellbeing (people in productive age and household heads) are also the ones who are privileged when decisions on intra-household resource allocation are to be made [55, 76]. Appraised in relation to the findings on children under five and women described earlier, these higher levels of OOPE among adults of productive age may indicate that free provision of services targeting this age group is not prioritized, leaving households to cope with substantial expenditures.

Similarly, it did not appear surprising that all measures of severity were associated with a higher OOPE. We wish to draw the reader’s attention to two particularly high coefficients, that related to hospitalization and that related to the presence of an underlying chronic condition (caused by communicable or not-communicable chronic diseases). These coefficients suggest that while the system may be capable of providing care free of charge as stipulated by its free healthcare policy for relatively simple conditions, it fails to do so in instances when this would be most needed, i.e., when an individual is so severely ill that they require hospitalization and/or suffers from an underlying chronic condition. Further studies are needed to look specifically into what clinical cases may result in such high OOPE. With the data at our disposal, we can only speculate that high OOPE for these specific episodes may be linked to drug and equipment shortages [12, 14, 67], forcing people to acquire necessities in private pharmacies. It should be noted that the financial burden we capture in our study for individuals suffering from chronic conditions is additional to the one captured by an earlier study by Wang et al. [23, 25, 45]. Their earlier study focused on individual expenditure for routine care for chronic non-communicable disease, while our study captures the additional financial burden faced by people who suffer from a chronic condition when seeking care for an acute illness episode. Appraising findings across the two studies jointly, we conclude that individuals suffering from a chronic condition receive very poor financial protection in the current system. This calls for the urgent implementation of policies specifically targeting their needs.

### Methodological considerations

This study represents one of the very first attempts made in Malawi to estimate OOPE on medical care in the context of a free healthcare policy while taking into account the selection bias that arises as such expenditure can only be observed among people who sought treatment at a health facility in the first place. The model statistics (distance to the closest health facility (km) p = − 0.091 and Wald test of independent equations (rho = 0) *p* < 0.001) confirm that the choice of the selection variable based on a conceptual understanding of the role of distance in shaping decisions to seek care [13, 20, 50–52, 55, 67, 82, 90, 91] was adequate.

Notwithstanding the strengths of our estimation model, our study is subject to several weaknesses. First, we need to be aware that, like many other prior studies [23, 50–52, 83], we relied on self-reported data collected retrospectively. Although we limited the relevant recall period to 4 weeks, we cannot exclude the possibility that individuals did not report the illness episode and its related healthcare seeking and expenditure accurately [92]. Second, our data did not differentiate OOPE across expenditure categories (e.g. drugs, laboratory tests, hospitalization or surgery), hence we cannot tell what items drove OOPE, and cannot provide more specific guidance for policy makers. Third, we must acknowledge the potential limitation that arises from the fact that our data date back to 2012/2013. To this regard, we wish to point at the fact that while the actual OOPE values might have changed over time, our analysis is not per se invalidated by the age of the data, given that our primary objective was showing persistence of high OOPE even in a context of free healthcare provision. Moreover, we trust that the overall policy environment has not changes dramatically since 2012 since no major large-scale health financing reforms aimed at enhancing financial protection have been implemented. Rather the opposite, due to shortages in donor funding, user fees have been temporarily reintroduced in selected settings and for selected services [93, 94]. In light of this, the OOPE values captured by our study are likely to be lower than current OOPE values. Studies based on more recent data are urgently needed to assess how OOPE and their determinants might have changed over time in light of the recent user fee reintroduction.

Furthermore, albeit not a weakness of our methodological approach per se since we purposely focused exclusively on estimating the financial protection afforded by the formal free healthcare system, the reader needs to consider that our OOPE values represent lower-bound estimates of total OOPE in Malawi. In pluralistic healthcare systems [95, 96], such as Malawi, individuals frequently move across types of care. To estimate total OOPE and not only OOPE related to formal healthcare use, one would therefore need to also account for expenditure on traditional treatments and/or for items purchased at a private pharmacy or on the market with no prior visit to a formal provider.

## Conclusion

Appraising findings from our study in relation to the broader literature on SSA indicates that OOPE for formal healthcare services in Malawi is relatively low compared with what is observed in other settings. Still, the free healthcare system in place is not sufficient to guarantee that all individuals effectively access care at no cost at point of use, since about one in four people continue to pay an average of 2.72 USD when seeking care. In addition, our findings also highlighted how only two out of three individuals sought formal care upon falling ill. Hence, considering together results from the primary and from the selection model, we conclude that while certainly being more effective than systems based on user charges, a system based on free healthcare provision does not provide an automatic guarantee that all ill individuals will receive the care they need and will do so facing no OOPE. Further measures, including observance of partnerships agreements (such as the SLAs) between the government and private institutions should be set in place to ensure that all citizens are granted access to the services stipulated in their legislation.

## Data Availability

The datasets used for the current study are not publicly available due standard procedures of the funding agency, but are available from the Principal Investigator, i.e., the last author, upon reasonable request.

## Abbreviations

MWK: Malawian Kwacha
OOPE: Out-of-pocket expenditures
CHAM: Christian Health Association
SLAs: Service Level Agreements
USD: United States Dollar
UHC: Universal Health Coverage
WHO: World Health Organization
SSA: Sub-Saharan African
