# Correction to: Determinants of healthcare seeking and out-of-pocket expenditures in a “free” healthcare system: evidence from rural Malawi

**DOI:** 10.1186/s13561-020-00277-w

**Published:** 2020-07-01

**Authors:** Meike Irene Nakovics, Stephan Brenner, Grace Bongololo, Jobiba Chinkhumba, Olivier Kalmus, Gerald Leppert, Manuela De Allegri

**Affiliations:** 1 Heidelberg Institute of Global Health, Faculty of Medicine and University Hospital, University of Heidelberg, Heidelberg, Germany; 2 Research for Equity and Community Health (REACH) Trust, Lilongwe, Malawi; 3 University of Malawi College of Medicine, Blantyre, Southern Region, Malawi; 4 German Institute for Development Evaluation (DEval), Bonn, Germany

**Correction to: Health Econ Rev 10, 14 (2020)**

**https://doi.org/10.1186/s13561-020-00271-2**

Following publication of the original article [1], the authors reported that the number “37.01” in Table 2 was moved up so it is now in the wrong line. The correct Table 2 is shown below. The original article [1] has been updated.

**Table 2:** Sample characteristics

		Acute ill sample (*n* = 2740)		Utilization formal care sample (*n* = 1884)		Positive out-of-pocket expenditures on medical treatment sample (*n* = 494)	
**Sample scale**		**N**		**N**		**N**	
	Number of villages	101		98		75	
	Number of households	1037		718		186	
**Individual level**							
		**Mean**	**SD**	**Mean**	**SD**	**Mean**	**SD**
Distance to the closest health facility (km)	continuous	2.22	1.25	2.19	1.24	2.14	1.28
Age (years)	continuous	21.43	18.2	19.56	17.57	19.62	17.12
Illness duration (days]	continuous	7.91	8.27	8.67	8.85	9.58	10.21
Accompanying persons	continuous			1.19	0.61	1.22	0.60
		**N**	**%**	**N**	**%**	**N**	**%**
Age	0 = 0–4 years;	513	18.72	419	22.24	117	23.68
	1 = 5–14 years;	793	28.94	562	29.83	133	26.92
	2 = 15–39 years;	971	35.44	637	33.81	171	34.62
	3 = 39+ years	463	16.90	266	14.12	73	14.78
Sex	0 = male;	1283	46.82	844	44.80	230	46.56
	1 = female	1457	53.18	1040	55.20	264	53.44
Education (education of household head if child < 14 years)	0 = no formal education	1726	62.99	1164	61.78	295	59.72
	1 = any formal education	1014	37.01	720	38.22	199	40.28
Household head	0 = others;	2157	78.72	1523	80.84	391	79.15
	1 = being household head	583	21.28	361	19.16	103	20.85
Reported chronic illness	0 = no chronic illness reported;	2356	85.99	1678	89.07	436	88.26
	1 = chronic illness reported	384	14.01	206	10.93	58	11.74
Limitation imposed on routine activities	0 = no perceived limitation on routine activities	1047	38.21	587	31.16	125	25.30
	1 = perceived limitation on routine activities	1693	61.79	1297	68.84	369	74.70
Hospitalization	0 = no hospitalization;	2636	96.20	1780	94.48	463	93.72
	1 = hospitalization	104	3.80	104	5.52	31	6.28
Socio economic status (wealth quartiles)	1 = poorest (1st wealth quartile)	686	25.04	484	25.69	108	21.86
	2 = poor (2nd wealth quartile)	685	25.00	486	25.80	109	22.06
	3 = less poor (3rd wealth quartile)	685	25.00	478	25.37	126	25.51
	4 = least poor (4th wealth quartile)	684	24.96	436	25.14	151	30.57
Household size	0 = 0–5 members;	1483	54,12	1036	54.99	297	60.12
	1 = 5+ members	1257	45,88	848	45.02	197	39.88
Location of household	0 = rural	2598	94.82	1791	95.06	470	95.14
	1 = urban	142	5.18	93	4.94	24	4.86

*SD* Standard Deviation
